# Isolation and Characterization of Infection of Four New Bacteriophages Infecting a *Vibrio parahaemolyticus* Strain

**DOI:** 10.3390/antibiotics13111086

**Published:** 2024-11-14

**Authors:** João Duarte, David Trindade, Vanessa Oliveira, Newton C. M. Gomes, Ricardo Calado, Carla Pereira, Adelaide Almeida

**Affiliations:** 1 Centre for Environmental and Marine Studies (CESAM), Department of Biology, University of Aveiro, 3810-193 Aveiro, Portugal; j.macedoduarte@ua.pt (J.D.); david.trindade@ua.pt (D.T.); v.oliveira@ua.pt (V.O.); gomesncm@ua.pt (N.C.M.G.); 2 Laboratory for Innovation and Sustainability of Marine Biological Resources of the University of Aveiro (ECOMARE), Centre for Environmental and Marine Studies (CESAM), Department of Biology, University of Aveiro, 3810-193 Aveiro, Portugal; rjcalado@ua.pt

**Keywords:** phage infection, bacterial inactivation, phage-resistant bacteria, bacterial fitness

## Abstract

Bacteria from genus Vibrio continue to be one of the most common threats to aquaculture sustainability. *Vibrio* spp. have been associated with infectious outbreaks in fish, shrimp, bivalves and even algae farms worldwide. Moreover, several *Vibrio* spp. are also pathogens that impact human health and are a threat to public health when transferred to consumers through contaminated seafood products. The use of bacteriophages is an evolving technology that could be applied in the treatment of *Vibrio* spp. either to protect aquaculture farms or to decontaminate seafood, namely bivalves during their depuration. In the present study, bacteriophages vB_VpS_LMAVpS1 (S1) vB_VpS_LMAVpVPP (VPP), vB_VpS_LMAVpSH (SH) and vB_VpS_LMAVpH (H) infecting *V. parahaemolyticus* were isolated and characterized. All phages presented fast adsorption rates and were able to control *V. parahaemolyticus* at all multiplicity of infections (MOIs) tested (MOI of 1, 10 and 100), with reductions of more than 4 log CFU/mL being recorded, but only in the presence of divalent cation calcium. The rate of emergence of phage-resistant mutants was very low (1.8 × 10^−6^ to 3.1 × 10^−6^). Bacterial phage resistance was not permanent and led to a loss of bacterial fitness. All four phages presented with lysins encoded in their genomes. The results presented provide valuable insights for future studies in the application of these bacteriophages in different scenarios to control, decontaminate or treat bacterial infections or contaminations of *V. parahaemolyticus*.

## 1. Introduction

Bacteria from the genus *Vibrio* are widespread and have been associated with numerous infectious disease outbreaks worldwide [1]. Among these, *V. parahaemolyticus* is the leading cause of seafood-related illness [2]. This microorganism can occur naturally in aquatic systems being found in saltwater, brackish and freshwater [1,3,4,5]. *Vibrio parahaemolyticus* can cause ulceration and hemorrhage in fish, leading to death and causing devastating losses to aquacultures [3,4,6]. Moreover, it has been observed that the bacterial concentration in animal tissue remains high even when it decreases in water [7]. This microorganism can be naturally present in the microbiota of other aquatic animals, such as shrimp, where they act as opportunistic pathogens [8,9], leading to mass mortality and detrimental losses of production [3,10,11]. For the bivalve industry, *V. parahaemolyticus* has also been considered an issue for concern, as it can also cause mass mortalities [12,13,14]. Furthermore, *V. parahaemolyticus* is considered the most common cause of seafood illness [15,16]. Consequently, this species poses a significant threat to multiple sectors related to farmed and wild seafood, as well as humans, thus being a critical one-health issue [5,16].

A substantial part of bivalves consumed worldwide are farmed in waters whose microbiological quality is often unfit for these organisms to be directly consumed by humans. In general, bivalves are required to be depurated prior to entering trade chains. Depuration facilities keep these animals in large recirculating water tanks which are sterilized through ultraviolet (UV) radiation, chlorine or ozone for several hours to days, depending on their levels of microbiological contamination [17]. In the case of *V. parahaemolyticus*, treatment can last from 2 days up to 15 days [17]. If not properly depurated, bivalves can pose a health risk to consumers, namely those species that are commonly eaten raw (e.g., oysters) [18].

Phage therapy uses bacteriophages (phages) to kill certain bacteria using viral natural life cycles [19,20]. This approach presents considerable benefits to the depuration industry, as phages have the ability to reach bivalve microbiota through their dispersal in the recirculating water [21,22,23]. Phage therapy can lead to bacterial inactivation deep within animals’ tissues. Furthermore, phages present high specificity towards bacterial targets and are normally unable to infect bacteria from different genera or species [23], hence having no considerable impact in bivalve’s microbiota and health during depuration [24]. Another advantage of phages is that during infection they will produce new virions, therefore increasing their concentration and increasing the success of reaching new bacterial targets [25]. As such, phages can be a possible solution to fight *Vibrio* spp. in numerous aquaculture scenarios, as well as during bivalve depuration [20,26]. However, for phage therapy to be successful, a continuous effort must be endeavored to isolate and characterize new bacteriophages to improve the understanding of viral–bacterial interactions. The different effects of phage to bacteria ratios (MOIs) must be carefully studied to determine the minimum phage concentration required to promote infection and the highest bacterial decrease [27]. In addition, divalent cation requirements (e.g., magnesium or calcium) [28] or phage sensitivity to environmental factors (pH, salinity and temperature of UV-radiation) [29] can also affect phages’ ability to promote infection. Therefore, only by gathering continuous information will researchers be able to formulate the best treatment or decontamination protocols and overcome bottlenecks, such as bacterial resistance [30].

In the present study, we used an environmentally isolated bacterial strain of *V. parahaemolyticus* as host for the isolation and characterization of four new *Vibrio* bacteriophages. The inactivation potential of the four phages was studied in-depth through the determination of divalent cation requirements and characterization of inactivation curves using different phage-to-bacteria doses. Additionally, bacterial resistance to phages was also studied and their impact on bacterial metabolic fitness was also assessed using phage-resistant and sensitive bacterial strains. The results of this study pave the way for future research in the application of phages for the inactivation of *Vibrio*.

## 2. Results

### 2.1. Phage Isolation and Virion Morphology

**Figure 1:**
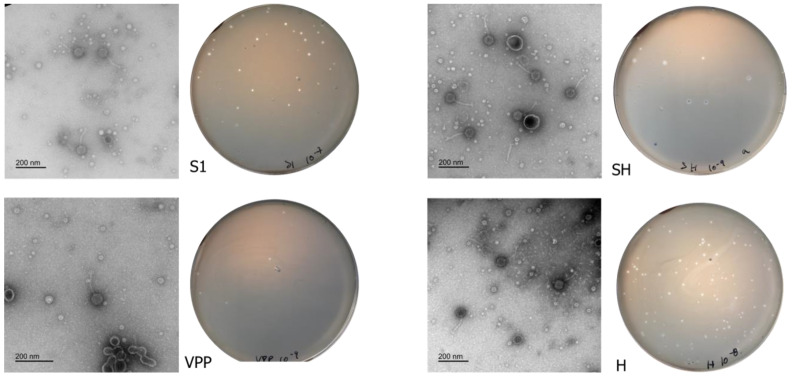
Phage virion morphology (**right**) and plaque morphology (**left**) of the four isolated phages.

Phages vB_VpS_LMAVpS1, vB_VpS_LMAVpVPP, vB_VpS_LMAVpSH and vB_VpS_LMAVpH (abbreviated to: S1, VPP, SH and H, respectively) formed clear plaques with strikingly different plaque morphologies. Phage S1 produced clear plaques (0.5 to 1 mm), Phage VPP and H produced small bulls-eye shaped plaques (0.5 mm), Phage SH produced large and clear plaques with a secondary turbid halo (1–1.5 mm plaque and halo between 0.5 and 1 mm) (Figure 1). Based on the morphological analysis by electron microscopy (Figure 1), all four phages exhibited siphovirus morphotypes and were classified as Caudoviricetes. Virions of phages S1, VPP, SH and H presented similar morphologies and varied only in size with icosahedral heads with approximately 56.13 ± 2.6, 72.94 ± 1.2, 60.39 ± 0.9 and 59.42 ± 1.0 nm width and tails with approximately 111.7 ± 2.7, 134.0 ± 3.0, 125.22 ± 1.1 and 104.26 ± 2.3 nm length, respectively.

### 2.2. Phage Host Range

All isolated phages presented a very narrow host range, infecting only the bacteria used as host for isolation (its host) of all the 26 bacterial strains tested (Table 1).

### 2.3. Phage Genome, Assembly and Annotation

The general information of the four phages is presented in Table 2. The genome analysis revealed that all phages belonged to the same family differing by less than 5% from each other at the nucleotide level using BLASTn; no significant alignment was found with any phage genome sequences deposited in GenBank (Table 3). All phages displayed a linear double-stranded DNA genome, with lengths ranging between 44,890 and 45,137 bp, and their GC content being 43.3% for S1 (GenBank: PQ284949) and VPP (GenBank: PQ284950), 43.4% for H (GenBank: PQ284952) and 43.5% for SH (GenBank: PQ284951) (Table 3). All phages were classified as complete with no evidence of host contamination. The S1, VPP and H genomes contained 66 predicted CDSs, with 42 and 43 CDSs predicted as hypothetical proteins (Table 2). Similarly, 67 CDSs were predicted in the genome of Phage SH, with 45 CDSs predicted as hypothetical proteins.

All predicted CDSs were categorized into six modules, including the DNA, RNA and nucleotide metabolism module, lysis module, head and packaging module, structure module (connector and tail), additional functions module (other), and a module for unclassified hypothetical proteins (Table A1, Table A2, Table A3 and Table A4). In all four genomes, our analysis did not identify any known genes associated with antibiotic resistance or virulence factors. Additionally, no integrase, anti-CRISPR proteins or tRNA genes were identified. The phage lifestyle was predicted to be virulent for all four phages by PhageAI [31].

**Figure 2:**
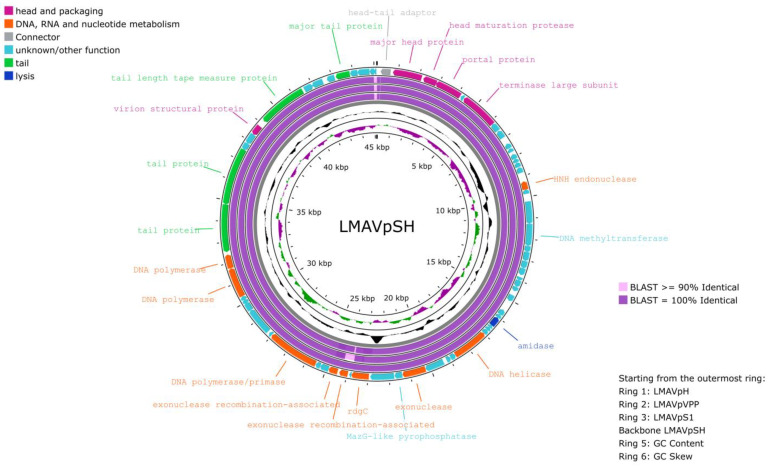
Proksee representation of the four phages isolated in this study. The genome of the SH phage was used as the reference for comparison with phages S1, VPP and H. The final circle with arrow-headed bands represents the coding DNA sequences (CDSs) of the SH phage color coded according to the functional category of the predicted gene in the direction of the transcription. The innermost ring represents the genome GC skew (green/pink) followed by GC content (black). The labels show the predicted functions of the functional CDS, color-coded by the PHROG category. The analysis was carried out on a PROKSEE Server that uses BLAST analysis to illustrate conserved and missing genomic sequences (accessed on 6 September 2024: https://proksee.ca/projects/new).

Comparative genomic analyses of the four phages isolated in this study revealed that regions with lower homology among them corresponded to a tail protein, a CDS encoding an exonuclease recombination-associated protein, and a hypothetical protein (Figure 2).

### 2.4. Phage Adsorption Curve

**Figure 3:**
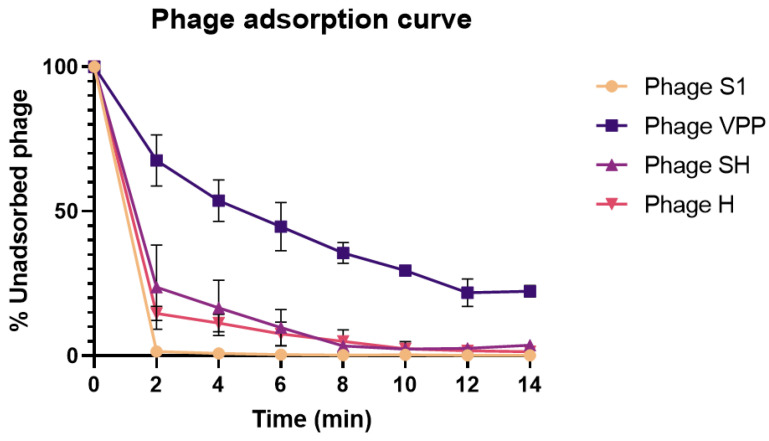
Phage adsorption curve. Samples were collected every 2 min. Three assays were performed for each phage. The results are expressed as the mean of three independent assays.

Phages S1, SH and H presented very fast adsorption to the host with less than 10% of the unadsorbed phage particles being detected after 4 min of incubation. Phage VPP presented a slower adsorption when compared with the remaining phages, with 50% adsorbed phages after 6 min (Figure 3). The constant of adsorption (k) was calculated for all phages after 6 min. Three phages showing very similar results: Phage S1: k = 7.17 × 10^−10^ mL^−1^ min^−1^; Phage SH: k = 3.06 *×* 10^−10^ mL^−1^ min^−1^ and Phage H: k = 3.39 × 10^−10^ mL^−1^ min^−1^. Phage VPP presented the slowest adsorption constant: k = 1.06 × 10^−10^ mL^−1^ min^−1^.

### 2.5. Evaluation of Calcium and Magnesium Requirements for Phage Replication

**Figure 4:**
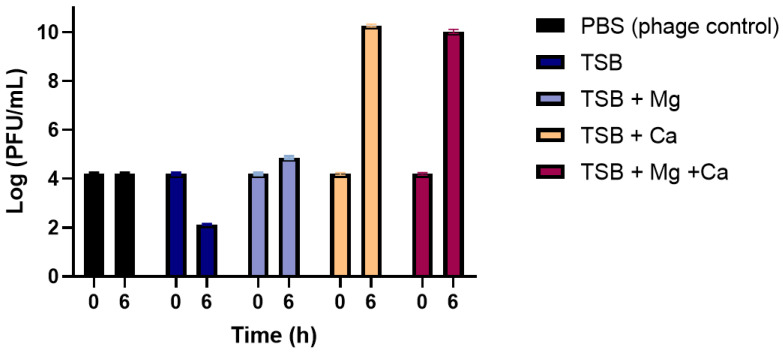
Phage SH concentration after 0 (left column) and 6 h (right column) of incubation in the presence of bacteria. PBS—phage inoculated in PBS without the presence of bacteria; TSB—phage inoculated in TSB in the presence of bacteria; TSB + Mg—phage inoculated in the presence of bacteria in media supplemented with magnesium; TSB + Ca—phage inoculated in the presence of bacteria in media supplemented with calcium; TSB + Mg + Ca—phage inoculated in the presence of bacteria in media supplemented with magnesium and calcium. All groups were inoculated with bacteria to a final concentration of 10^5^ CFU/mL and phage to a final titre of 10^4^ PFU/mL. The results are expressed as the mean of three assays.

Phage virion production varied significantly between the different test groups (*p* < 0.05). After 6 h of incubation, phage titre had increased to 10.27 log (PFU/mL) and 10.02 log (PFU/mL) in the group supplemented with calcium and with calcium and magnesium, respectively. In the presence of magnesium and the absence of calcium, phage titre had slightly increased to 4.8 Log PFU/mL (about 0.7 log increase). However, in the absence of both divalent cations (group TSB 3% NaCl) phage titre had decreased about 2 log (final titre of 2.12 Log (PFU/mL). The phages from the control group (PBS) remained stable (Figure 4).

#### Evaluation of Phage Infection in Media with Different Concentrations of Ca^2+^

**Figure 5:**
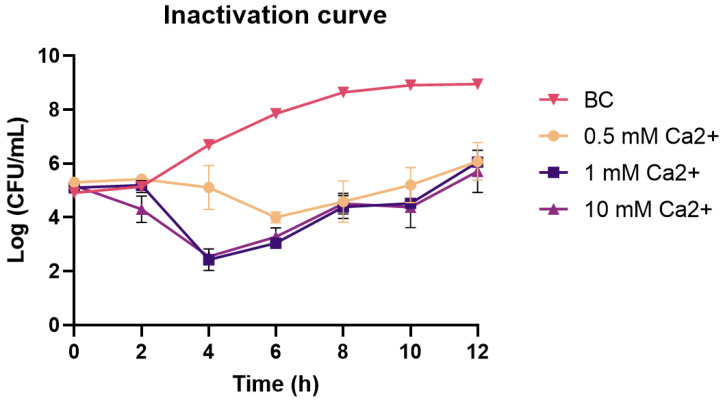
Bacterial inactivation curves by Phage SH in medium supplemented with different concentrations of calcium. All groups were inoculated with the same concentrations of phage and bacteria. Bacterial control (BC)—bacteria inoculated without the presence of phages; 0.5 mM Ca—phage and bacteria in media supplemented with 0.5 mM of calcium; 1 mM Ca—phage and bacteria in media supplemented with 1 mM of calcium; 10 mM Ca—phage and bacteria in media supplemented with 10 mM of calcium. The results are expressed as the mean of three independent assays.

**Figure A1:**
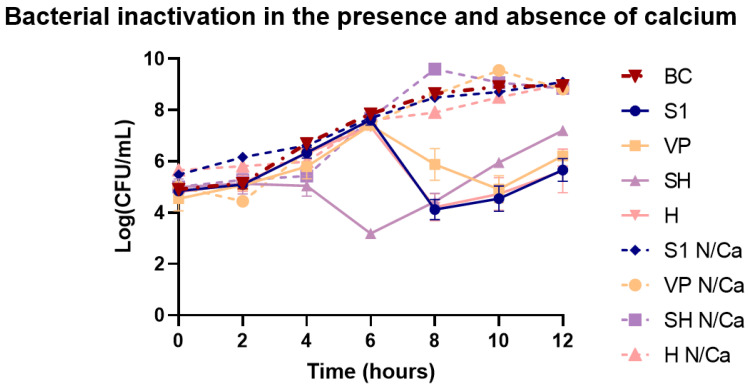
Inactivation curves of the four phages in standard media and in media supplemented with calcium to a final concentration of 1mM. BC—Bacterial control: bacteria grown in TSB supplemented with calcium and in the absence of any phages. S1, VP, SH and H: bacteria inoculated in the presence of the specific phages in media supplemented with calcium to a final concentration of 1 mM. S1, VP, SH and H N/Ca: bacteria inoculated in the presence of the specific phages in standard media with no calcium added.

After determining the phages divalent cation requirements, to better understand the effect of calcium in phage infection, three kill curves were performed under varying concentrations of calcium (Figure 5). The bacterial inactivation in the medium supplemented with Ca [1–10 mM] resulted in significantly higher inactivation when compared with the inactivation obtained in medium supplemented with Ca [0.5 mM] (*p* < 0.05). However, there were no significant differences between the media supplemented with 1 mM or 10 mM (*p* > 0.05). Using a concentration of 1 mM, another inactivation curve was performed for all phages individually which was compared with the inactivation curve of the same phages in the absence of calcium (Figure A1).

### 2.6. Bacterial Kill Curves

The rate of bacterial inactivation was determined for all phages individually and using an MOI of 1, 10 and 100.

**Figure 6:**
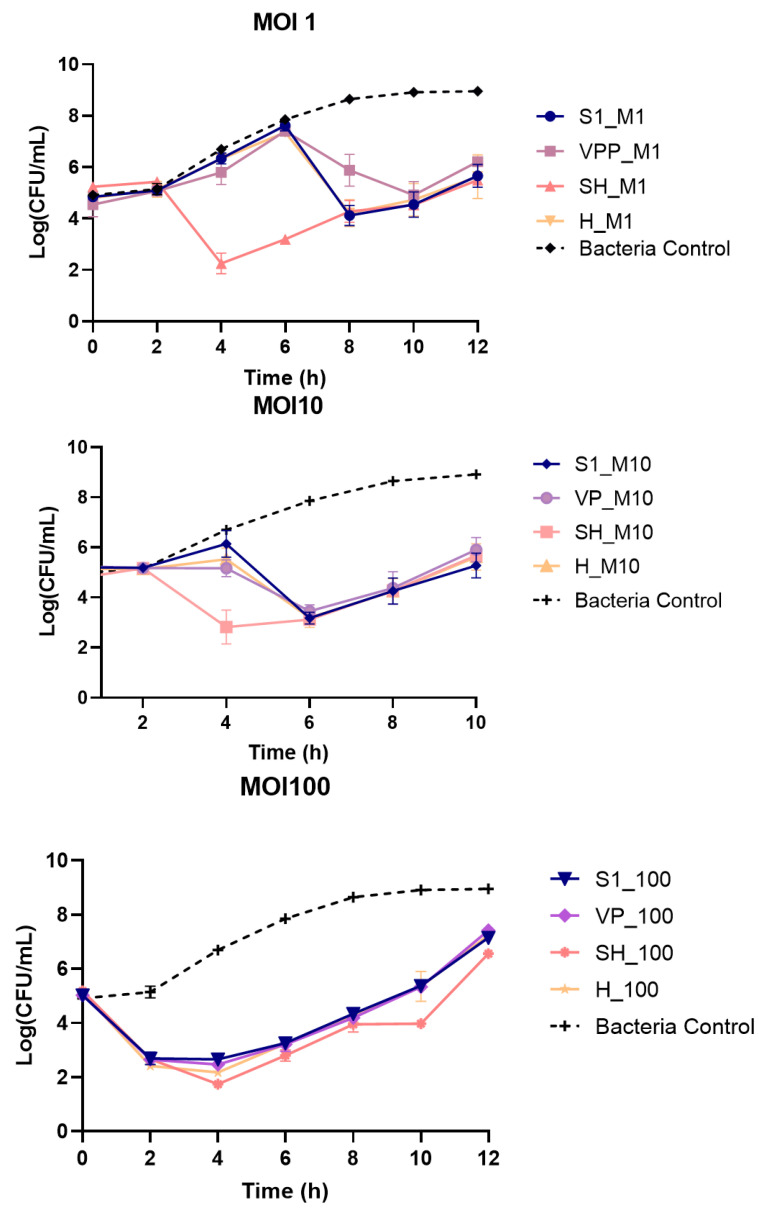
Bacterial inactivation curves at different MOIs. Bacterial control—bacteria incubated in the absence of phages. S1, VP, SH and H represent the bacterial inactivation curves in the presence of the respective phage at an MOI of 1 (**top**), 10 (**middle**) and 100 (**bottom**). The results are expressed as the mean of three independent assays.

At an MOI of 1, Phage S1 and Phage H presented similar inactivation curves, reaching their maximum inactivation at 8 h (4.5 ± 0.2 log CFU/mL) and with no significant differences being recorded during the whole assay (*p* > 0.05). Phage VPP at an MOI of 1 inactivated the bacterium after 6 h of incubation, reducing the bacterial concentration until the 10th h of incubation (Figure 6), reaching a maximum inactivation of 4.0 log CFU/mL. Phage SH presented the fastest inactivation, starting after 2 h and continuing to decrease until the sixth h with a maximum inactivation of 4.7 log CFU/mL. After 8 h of incubation, Phage SH presented no significant differences when compared with those of Phage S1 and Phage H. However, at the end of the assay, the bacterial regrowth in the presence of Phage SH was significantly higher than that of the remaining phages (*p* < 0.05).

When phage concentration was increased (MOI 10), the inactivation by all phages began sooner (Figure 6). With the increase in phage concentration, inactivation curves for Phages S1, VPP and H became very similar (Figure 6), with no significant differences being detected (*p* > 0.05). Nonetheless, the maximum inactivation for all three phages was enhanced by the increase in phage titre, to 4.7, 4.4 and 4.7 CFU/mL for Phages S1, VPP and H, respectively, after 6 h. Phage SH reached its maximum inactivation after 4 h of incubation with 4.7 log CFU/mL reduction and was significantly different from the that of the remaining phages (*p* < 0.05).

At an MOI of 100, all inactivation curves became very similar with bacterial decrease being observed after 2 h (Figure 6). No significant differences were observed between Phages S1, VPP and H (*p* > 0.05). However, after 4, 6, 10 and 12 h of incubation, the differences between the Phage SH curve and those of the remaining phages were statistically significant (*p* < 0.05).

**Figure 7:**
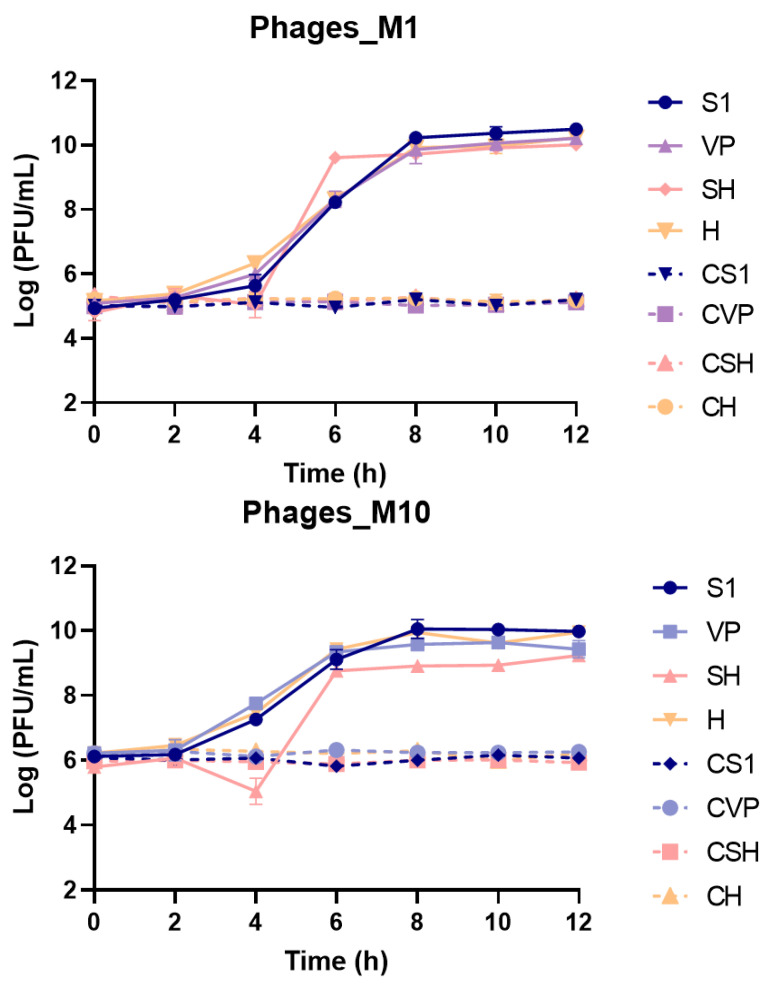

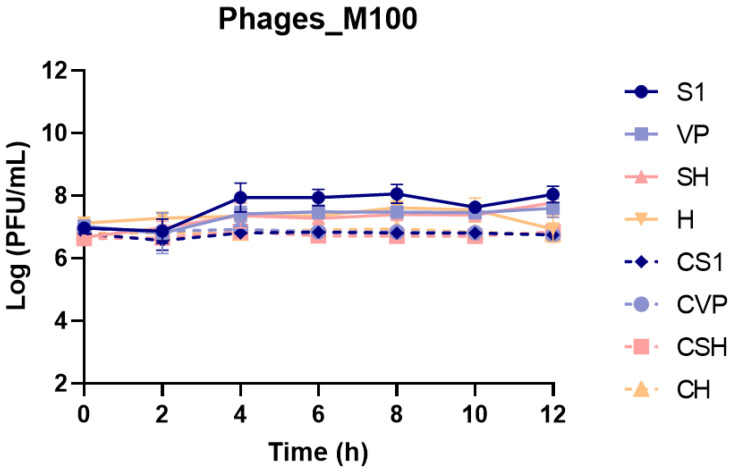
Phage virion production and phage control during incubation with bacteria. Three different MOIs were evaluated: 1 (Phages_M1 (**top**)), 10 (Phages_M10 (**middle**)) and 100 (Phages_M100 (**bottom**)). S1, VP, SH and H represent the phage groups incubated in the presence of bacteria. CS1, CVP, CSH and CH represent the phage control groups incubated in the same conditions but without any host present. The results are expressed as the mean of three independent assays.

During all assays, phages remained stable in the control group with no significant titre variations (Figure 7). At an MOI of 1 all phages significantly (ANOVA, *p* < 0.05) increased their titre to final concentrations of about 10 log PFU/mL (Figure 7). However, with the increase in MOI, phages produced less virions, reaching a final titre of 9.2–9.9 log PFU/mL at an MOI of 10 and 7.3 to 7.9 log PFU/mL at an MOI of 100. Phage SH at an MOI of 10 presented significantly lower virion production when compared with other phages (ANOVA, *p* < 0.05). At the highest concentration (MOI 100), Phage S1 produced more virions than the other phages with variations in titre being significantly different after 6 h when compared with that of Phages SH and H (*p* < 0.05) and after 8 h for Phages VP and SH (*p* < 0.05). However, by the end of the assay, due to a decline in phage titre, only Phage H presented significantly lower titre (*p* < 0.05). For all phages, statistically significant differences were observed between all infection groups when compared with their respective control (*p* < 0.05) suggesting a positive infection with virion production in all test groups.

### 2.7. Rate of Emergence of Phage-Resistant Bacterial Mutants

When *V. parahaemolyticus* 022C was exposed to the four phages, resistance developed similarly (Table 3). Moreover, the differences observed for the frequency of mutation were not statistically significant (ANOVA, *p* > 0.05).

**Table 3:** Frequency of spontaneous phage-resistant mutants of *V. parahaemolyticus* when exposed to single suspensions of phage.

Phage	Control Sample (CFU/mL)	Sample Treated with Phages	Frequency of Mutation
S1	1.13 ± 0.17 × 10^10^	1.98 ± 0.55 × 10^4^	1.75 × 10^−6^
VPP	1.13 ± 0.17 × 10^10^	3.50 ± 0.61 × 10^4^	3.09 × 10^−6^
SH	1.13 ± 0.17 × 10^10^	2.04 ± 0.52 × 10^4^	1.81 × 10^−6^
H	1.13 ± 0.17 × 10^10^	1.67 ± 0.41 × 10^4^	1.48 × 10^−6^

### 2.8. Study of Bacterial Resistance to Phage

#### 2.8.1. Loss of Bacterial Resistance to Phage

After 10 consecutive isolations and incubation in liquid, only one of the ten initial bacterial conolies had lost its phage resistance with a positive infection on the three isolated colonies. On the ninth passage, the resistant isolate ten revealed a loss of resistance with two colonies forming a lysis area. However, of the three isolated colonies tested, one of them remained clearly resistant with no signs of lysis when the spot test was performed. Nontheless, on the following passage (10th) all isolated colonies were phage sensistive.

The results are summarized in Table 4.

#### 2.8.2. Evaluation of Bacterial Fitness and Bacterial Resistance to Phages

**Figure 8:**
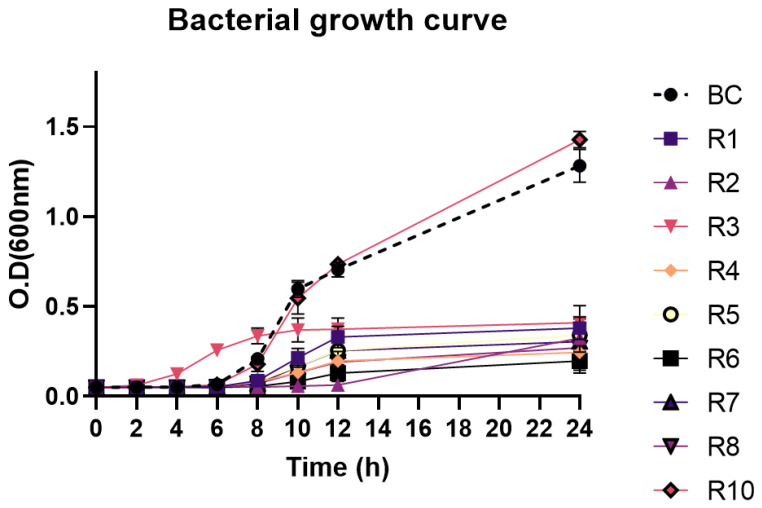
Growth curve of phage-resistant bacterial mutants and sensitive bacteria during 24 h using optical density readings at 600 nm.

The growth curve of the 10 strains from the previous passages (9 resistant and 1 sensitive) was compared with the growth curve of the original strain (Figure 8). After 8 h of incubation, significant differences were observed between the nine resistant strains and the control group. After 12 h, all resistant strains were already revealing slower growth curves than the control group. Notably, the strain that had lost phage resistance (R10) revealed a growth curve similar to the original strain.

## 3. Discussion

Infections caused by *V. parahaemolyticus* are a cause for concern in aquaculture systems and pose a risk to seafood consumers due to their pathogenicity, highlighting the need for alternative treatment or decontamination protocols in the sector [32,33]. Phage therapy can be used to treat vibriosis in farmed animals and can also be used to improve the microbiological safety of seafood. Thus, the continuous isolation and characterization of different phages is required to formulate the best treatment protocols, as well as to improve our current understanding of the many factors affecting phage infection. In the present work, we isolated four different phages and studied their infection in order to properly characterize the condition in which the highest virion production and the highest bacterial mortality will be observed.

After the isolation of the four bacteriophages, a loss of virions was observed when these were incubated in the presence of the host (Figure A1). Consequently, prior to studying bacterial defense mechanisms, the requirements for successful phage infection in the presence of divalent cations were determined. Phage SH was used as a model in these experiments, as the remaining three phages under the tested conditions were unable to cause bacterial lysis, and in this preliminary stage of work, the possibility of phages being temperate could not be excluded. Landry et al. [28] have also characterized the importance of divalent cations for a successful phage infection and observed that in the absence of calcium, the efficiency of plating was reduced in 98%. Similarly, in this study it was determined that calcium was required to detect virion production and in its absence phage titre would decrease (Figure 4). Magnesium has also been considered an important cation for phage’s replication and attachment [34,35]. However, for these specific phages, it was observed that phage attachment was not magnesium dependent, as a significant decrease in phage titre was observed when phage was incubated with bacteria. An increase of only 0.7 log in phage titre was recorded when the incubation was performed in the same conditions in the presence of magnesium. A similar reduced efficiency was also observed when Ba^2+^, Sr^2+^, Mg^2+^ or Mn^2+^ was used in the absence of Ca^2+^ in the study conducted by Landry et al. [28]. It is possible, in our study, that phages were able to recognize bacterial receptors but the injection mechanism was defective in the absence of magnesium [34], thus leading to a reduction in viable phage particles in its absence. However, when magnesium was added, a very small increase in phage virions was recorded. An indication of post-adsorption dependence of another divalent cation (calcium). Nonetheless, further studies are required to shed light on the observed phenomena.

As it was determined that calcium was required for a proper phage infection, the concentration of 1 mM was selected for further experiments, as using medium supplemented with concentrations of 1 mM and 10 mM of calcium presented a similar inactivation potential. It was observed that in the absence of calcium, bacterial growth was similar to that of the bacterial control group (Figure A1). Therefore, it was assumed that the same requirements would be valid for the four phages, and all the subsequent experiments were carried out in medium supplemented with 1 mM of calcium. Magnesium to a final concentration of 1mM was also maintained. This concentration is in line with the range of suggested concentrations in the literature (0.5 to 10 mM of Ca^2+^ or Mg^2+^) [36,37].

The four phages presented a very similar genome size (range 44.890–45.133 bp) and none of the phages contained known genes associated with virulence, integrases or antibiotic-resistance genes. Furthermore, the potential lack of integrase genes suggests that the phages were strictly lytic. Overall, the phage genetic analyses indicated that they are not closely related to any known sequences in GenBank, suggesting that they may belong to a new phage family. Additionally, we detected phage lysins in the genome of all phages, a feature which may be of biotechnological importance due to their ability to cause bacterial inactivation [38]. Lysins could be purified for direct application, or the genes encoding these lysins could be used to genetically engineer industrially domesticated microbes, thus expanding the therapeutical applications and industrial possibilities for these phages. Despite genetic similarities, distinct phage plaque morphologies were observed for the four phages, and through electron microscopy analysis we observed that phage virion presented some morphologic differences. Furthermore, genetic analysis revealed that they presented differences in regions coding for a tail protein, an exonuclease and in a hypothetical protein. The phage tail proteins are involved in host recognition and penetration [39], and exonuclease may have an important role during replication [40]. These differences may lead to the morphological and infective profile of each isolated phage.

Phage host specificity is considered an advantage of phage therapy. In the present study, none of the isolated phages were able to infect the other non-host bacteria tested. This may pose as a disadvantage to future applications in aquacultures or depuration units since the likelihood that an animal is infected with a given specific bacterium is very low. However, isolating phages with a broad host range seems to be a very challenging task [41]. Tan and colleagues [42] isolated 6 different *V. parahaemolyticus* phages and their host range revealed that they could only infect 18.3% of all *V. parahaemolyticus* strains tested (126 strains). In a similar study, phage VP06 was able to infect 28.1% of the strains tested, thus showing a higher infectivity towards other environmental strains [43]. In the future, the phages reported in the present work need to be combined with other phages to produce phage cocktails, thus expanding their host range. On the other hand, the development of experimental protocols for phage co-evolution can be a possible solution to obtain new phages with broader host ranges within the genus *Vibrio*. Since the mainstream isolation procedure does not always result in increased outcomes of successful isolation, post-isolation manipulation could be a better alternative to broader host ranges. Different studies have already proven the ability to enhance phage host range through the application of Appelman’s protocol or similar adaptation protocols [44,45].

Phage adsorption to the bacterial cell is an important factor for infection, as it directly influences the phage’s ability to kill the bacteria [27]. In our study, Phage S1, SH and H were able to rapidly adsorb to bacterial cells within 2 to 4 min. When phages are added to suspensions containing the bacterial hosts, more than a single phage particle can irreversibly adsorb to a cell [27]. To guarantee that more bacterial cells are infected simultaneously, increasing phage concentrations can be an option. However, the increase in phage ratio may result in a phenomena called lysis from without [46] and can negatively impact therapeutical outcomes. Therefore, it is important to study the impact of phage–bacteria ratios in order to design adequate protocols that guarantee a threshold for the best possible outcomes. In our study, phages were able to control *V. parahaemolyticus* in vitro regardless of the MOI used. However, an increase in MOI resulted in a faster bacterial inactivation, which is an important observation for scenarios where faster treatments are needed, such as during bivalve depuration. Furthermore, the bacterial concentration reached lower values when higher phage doses were used for all phages. Despite the fast adsorption rate observed, at the highest MOI used we observed very low virion production, indicating that the reduction in bacterial concentration may be affected by other factors, such as lysins or lysis from without [46]. This indicates that, for a decontamination protocol aiming at inactivating the maximum bacteria possible during short periods of time, using high concentrations of phage could be a promising alternative. Similar observations have been described by several researchers for different bacteria [47,48,49,50,51,52]. In a similar study to characterize two different phages for *V. parahaemolyticus*, Liang and colleagues [52] observed that increasing MOI from 0.01 to 10 resulted in a prolonged control of bacterial growth. However, after 6 h of incubation the bacterial regrowth was higher in the groups that had more phage added. Furthermore, after 20 h of assay, an MOI of 0.01 presented the lowest bacterial regrowth. Bacterial phage-resistant mutants are expected to emerge during phage exposure leading to regrowth curves [29]. In the present study, bacterial regrowth was observed in all MOI tested.

The rate at which bacteria will become phage resistant is both dependent on the type of phage and its receptors and the type of bacteria [53]. The calculation of bacterial phage-resistant mutation rates indicated similar mutation rates for all phages tested in this study. However, mutation rates were considerably lower than those previously observed for other phages [23,29,54,55]. We did not infer the potential of combining these phages in cocktails due to their similarity. In the future, using new isolated phages capable of infecting this *V. parahaemolyticus* strain could improve control and avoid the growth of resistant mutants by application of different phages sequentially [56]. Nonetheless, with such a low frequency of mutation, the potential for application of any of the isolated phages should not be hindered. Moreover, these assays were performed in rich nutrient media allowing for the resistant mutants to grow. This scenario is not expected to occur in aquaculture or depuration facilities.

Most phage resistance comes at a cost which varies for each bacteria [57]. Changes in growth rate, bacterial motility, antibiotic resistance or virulence can be observed in phage-resistant bacterial mutants [58,59,60]. Therefore, studying the impact of an isolated phage in the target strain can help to understand bacterial resistance mechanisms and provide valuable insights for other therapeutical approaches such as antibiotics [61]. In this study, one of our isolated phages (S1) was used to understand the impact of bacterial resistance. The use of this phage was decided because it presented the clearest phage plaques with no indication of possible formation of lysins that could influence our results. Initially, the resistant mutants were isolated and ten consecutive passages were performed to understand if the resistant bacteria would revert back to their susceptible type. Although 9 out of the 10 isolates remained bacterial resistant, we observed a progressive loss of phage resistance in 1 of the isolates. Other studies have also observed a loss of phage resistance after 3–5 passages [62,63]. However, despite not having observed a loss of resistance in all isolates, phage-resistant mutants presented slower growth curves reaching clearly inferior optical densities when compared with the parental strain. Furthermore, the only isolate that completely lost phage resistance presented a growth curve very similar to the control group. Indeed, if phages can remain viable during prolonged applications, several bacterial multiplication cycles may take place, and in the absence of bacterial regain of susceptibility, the cost in bacterial fitness may still provide favorable outcomes.

## 4. Materials and Methods

### 4.1. Bacterial Strains and Growth Conditions

The bacterial strains used in this study are listed in Table 2. The bacterial strain *Vibrio parahaemolyticus* O22C was used as the phage host in this study. *Vibrio parahaemolyticus* ATCC 17802, *V. vulnificus* ATCC 19.1, *V. vulnificus* ATCC 27562, *Aliivibrio fisheri* ATCC 49387, *Aeromonas hydrophila* ATCC 7966, *Escherichia coli* ATCC 13706, *V. alginolyticus* CECT 521, *A. salmonicida* CECT 894, *V. parahaemolyticus* DSM 27657 and *Photobacterium damselae* DSM 7482 were purchased from ATCC, CECT and DSM collections. *Vibrio* VL06, *Vibrio* VL018, *Vibrio* CF02, *Vibrio* CF03, *Vibrio* CF06, *V. parahaemolyticus* V10A, *V. parahaemolyticus* V9A, *V. parahaemolyticus* V9B and *V. parahaemolyticus* O22C were environmentally isolated from water samples, as already described in a previous study [64]. *Pseudomonas aeruginosa* was isolated from a hospitalized patient. Fresh bacterial cultures were kept at 4 °C in Tryptic Soy Agar (TSA, Liofilchem, Roseto degli Abruzzi, Italy) supplemented with 3% NaCl for marine bacteria. Before each assay, one isolated colony was transferred to 30 mL of Tryptic Soy Broth (TSB, Liofilchem, Roseto degli Abruzzi, Italy) and grown overnight at 25 °C or 37 °C with orbital shaking (120 rpm) until an optical density (O.D. 600) of 0.8 was achieved, corresponding to approximately 10^9^ cells mL^−1^.

### 4.2. Phage Isolation and Purification

Phages vB_VpS_LMAVpS1, vB_VpS_LMAVpVPP, vB_VpS_LMAVpSH and vB_VpS_LMAVpH (abbreviated to: S1, VPP, SH and H, respectively) were isolated from water samples collected at Ria de Aveiro estuarine network irrigation channels of the *Salinas da Marinha da Noeirinha*, an active marine salt production facility, and from Aveiro fishing harbour. About 50 mL of water were filtered through 0.45 µm pore size polycarbonate membranes (Millipore, Bedford, MA, USA) and added to 50 mL of a twice concentrated TSB medium. The mixture was inoculated with 1 mL of *V. parahaemolyticus* O22C in exponential phase and incubated for 24 h at 25 °C under 120 rpm. After incubation, the solution was centrifuged at 10,000× *g* for 10 min at 4 °C and filtered through a polyethersulfone membrane with a 0.22 µm pore size (Merck-Millipore, Darmstadt, Germany). The filtrate was stored at 4 °C and titre was determined according to [65]. Serial dilutions of the filtrate stock were performed in phosphate buffer saline (PBS) (137 mmol L^−1^ NaCl (Sigma, St. Louis, MO, USA), 8.1 mmol L^−1^ Na_2_HPO_4_·2H_2_O (Sigma, St. Louis, MO, USA), 2.7 mmol L^−1^ KCl (Sigma, St. Louis, MO, USA), and 1.76 mmol L^−1^ KH_2_PO_4_ (Sigma, St. Louis, MO, USA), pH 7.4). Five hundred microliters of each dilution, along with 200 µL of fresh bacterial culture, were added to 5 mL of molten TSB 0.6% top agar (30 g/L TSB (Liofilchem, Roseto degli Abruzzi, Italy), 6 g/L agar (Liofilchem, Roseto degli Abruzzi, Italy), 0.12 g/L MgSO_4_ (Sigma, St. Louis, MO, USA), and 0.05 g/L CaCl_2_ (Sigma, St. Louis, MO, USA), pH 7.4) and poured over a TSA plate. Plates were incubated at 25 °C and observed for the presence of lytic plaques after 16 to 18 h. One single plaque was selected and transferred to a 2 mL tube containing PBS and was homogenized. Subsequently, the suspension was serially diluted and plated over the double agar layer. Three successive single-plaque isolation cycles were performed in order to acquire pure phage stocks. All lysates were centrifuged at 10,000× *g* for 10 min at 4 °C, to remove bacteria or bacterial debris. Phage suspensions were kept at 4 °C.

Phage stocks were prepared by adapting the protocols described by Adams M. [65]. Three hundred µL of fresh exponential bacterial culture were transferred to 5 mL of molten top agar (3% NaCl) and poured over a TSA plate supplemented with 3% NaCl. After the plates were allowed to dry, 100 µL of the previously prepared stock was inoculated in the center of the plate over the bacterial lawn. A total of 10 plates were produced. The plates were incubated at 25 °C for 16–18 h until a clear lysis had formed on the center. The area where lysis occurred was collected and transferred to an Erlenmeyer flask containing 50 mL of SM buffer (0.1 M NaCl (Sigma-Aldrich, St. Louis, MO, USA), 20 mM Tris-HCl (Sigma, St. Louis, MO, USA) and 8 mM MgSO_4_ (Sigma, St. Louis, MO, USA), pH 7.5). The solution was agitated for 6 h at 80 rpms to allow phages to resuspend in the buffer. Subsequently, the solution was centrifuged at 10,000× *g* for 10 min at 4 °C to remove bacteria and agar debris and filtered through a polyethersulfone membrane with a 0.22 µm pore size. The final titre was confirmed through a double agar layer.

### 4.3. Electron Microscope Examination

Phage particles from high titre stock suspensions (10^10^ to 10^11^ PFU/mL) were negatively stained with 2% uranyl acetate (Electron Microscopy Sciences, Hatfield, UK) and subjected to electron micrographs using a JEOL 1011 transmission electron microscope (JEDL USA Inc., Peabody, MA, USA) operating at 100 kV. The images were obtained with a Gatan CCD–Erlangshen ES100W.

### 4.4. Phage Host Range

The phage host range was assessed for the bacterial strains listed in Table 2. through spot testing according to Adams M. [65]. Briefly, 300 µL of bacterial cultures in exponential phase were added to 5 mL of molten top agar, overlayed on TSA plates and allowed to dry. Subsequently, 10 to 50 µL of phage stock were poured over the bacterial lawn and plates were incubated at 25 °C for 16 to 18 h. A clear lysis zone at the spot determined bacterial sensitivity to the phage. Bacteria were differentiated according to either a clear lysis zone (+) or no lysis zone (−) (Table 2). The efficiency of plating (EOP) was determined for bacteria with positive spot tests (occurrence of a clear lysis zone), using the double-layer agar method. The EOP was calculated (average PFU on target bacteria/average PFU on host bacteria) [66] along with the standard deviation for the three measurements. For each phage, three independent assays were performed.

### 4.5. Phage Adsorption

The phage adsorption curve was determined according to Hyman and Abedon [67]. Briefly, phages (titre of about 10^5^ PFU/mL) were added to a bacterial culture (cell density of about 10^9^ CFU/mL) at 25 °C in TSB. Every 2 min, an aliquot was transferred to a tube containing 10% (*v*/*v*) chloroform, and the sample was centrifuged at 10,000× *g* for 1 min at 4 °C. The number of unadsorbed phage particles was determined by the double agar layer technique [65].

The percentage of adsorbed phages was calculated by comparing the free phage titre at a given point with the initial phage titre. Three independent assays were performed. The adsorption constant was calculated according to Zurabov et al. [68] using the following formula:

$$k=\left(-\frac{1}{Bt}\right)x\ln\left(\frac{P}{P_{0}}\right)$$

*P* represents the concentration of free phage per mL, *P*_0_ represents the initial concentration of phage, *B* represents the initial concentration of bacteria, *k* is the adsorption rate constant (mL/min) and *t* represents time (min).

### 4.6. Phage Genome, Assembly and Annotation

Phage genome sequencing was conducted by SeqCenter, LLC. (Pittsburgh, PA, USA). Illumina sequencing libraries were prepared using the tagmentation-based and PCR-based Illumina DNA Prep kit with custom integrated DNA technology (IDT) 10 bp unique dual indices (UDI) targeting an insert size of 320 bp. The quality of raw sequencing reads was assessed using FASTQC v0.12.1 (FastQC source: Bioinformatics Group at the Babraham Institute, Cambridge, UK). Low-quality reads were then trimmed with Trimmomatic v0.39, using the parameters: ILLUMINACLIP.fasta:2:30:1, Leading:8, Trailing:8, Slidingwindow:4:15 and Minlen:100, as described by Bolger et al. (2014) [69]. The processed reads underwent de novo genome assembly using SPAdes v3.15.5 with the-careful parameter [70]. The resulting assembly graph was visualized with Bandage v0.8.1 [71]. To evaluate coverage, the reads were mapped back to the assembled genome using BBMap v38.18 [72]. Contigs with inconsistent coverage were manually filtered out. The reads were remapped to the filtered contigs using Bowtie2 v2.5.1 [73] and reassembled with SPAdes. The packaging mechanisms and genome termini were determined using PhageTerm [74]. The genomes were identified as circularly permuted using apc.pl (https://github.com/jfass/apc, accessed on 22 November 2023). Further error correction and polishing of the genome sequences were performed with Pilon v1.24 [75]. The full-genome sequences of the four phages were compared to known phage sequences in GenBank using BLASTN to identify closely related phages. The completeness and contamination levels of the genome sequences were evaluated using CheckV v1.0.1 [76]. The genomes were annotated for coding DNA sequences (CDS), tRNA, tmRNA, CRISPRs, virulence factors, toxins and antimicrobial resistance genes using Pharokka v1.3.2 [77]. The CDS, were functionally categorized using PHROGs [78]. The phage lifestyles and phylogeny were classified with the AI-powered platform PhageAI v1.0.2 (https://phage.ai/).

### 4.7. Evaluation of Calcium and Magnesium Requirements for Phage Replication

Bacteriophage infection may require divalent cations, such as calcium or magnesium [28,65]. Therefore, it is important to characterize phages’ requirements for an optimal infection prior to any further testing. To investigate the phages’ requirements for successful infection, the virion production of one of the phages (phage VpM-SH) in the presence and absence of Ca^2+^ and Mg^2+^was compared during 6 h. Six different groups were established for this test (Table 5): Phage control PBS—PBS and phage; Phage control TSB—TSB 3% NaCl and phage; Infection group TSB—TSB 3% NaCl inoculated with bacteria and phage; Infection group Calcium—TSB 3% NaCl supplemented with 1mM Ca^2+^ and inoculated with bacteria and phage; Infection group Magnesium—TSB 3% NaCl supplemented with 1 mM Mg^2+^ and inoculated with bacteria and phage and the last group Infection group Calcium and Magnesium—TSB 3% NaCl supplemented with 1 mM Ca^2+^ and 1 mM Mg^2+^ and inoculated with bacteria and phage. To prepare the solutions, calcium chloride (FlukaTM Honeywell, Wabash, IA, USA) and magnesium sulphate (Sharlau, Sentmenat, Spain) were added to the TSB media to a final concentration of 1 mM. All groups were inoculated with phage to a final titre of 10^4^. Bacteria was added to a final concentration of 10^5^ CFU/mL.

#### Characterization of Phage Infection in Varying Concentrations of Calcium Cation

To better understand the impact of calcium concentration in phage infection, an infection curve was performed for Phage SH using varying concentrations of calcium [0.5–10 mM] Ca^2+^ in TSB 3%NaCl. To form the three different test groups, calcium chloride (Fluka^TM^ Honeywell, Wabash, IA, USA) was added to the TSB to a final concentration of 0.5 mM, 1.0 mM and 10 mM. Sterilized glass Erlenmeyer flasks with 30 mL of TSB + 3%NaCl + [0.5–10 mM] Ca^2+^ were inoculated with bacteria (final concentration of 10 CFU/mL) and phage (final concentration of 10^5^ PFU/mL) and incubated for 12 h without agitation. Additionally, two control groups were added: Phage control—phage inoculated in the absence of bacteria and Bacteria control—bacteria inoculated in the absence of phage. Samples were collected every 2 h during the 12 h. The phage titre was determined in triplicate by the double-layer agar method after an incubation period of 16 to 18 h at 25 °C. Bacterial concentration was determined in triplicate in solid TSA medium through the Miles and Misra method [79].

### 4.8. Bacterial Kill Curves

Bacterial kill curves were determined for *V. parahaemolyticus* O22C over 12 h for all phages at MOIs of 1, 10 and 100. To obtain the specific MOI, sterilized glass Erlenmeyers with 30 mL of TSB medium with 3% NaCl and 1mM of Ca^2+^ were inoculated with exponential bacterial culture (final concentration of 10^5^ CFU/mL) and phage suspension (final concentration of 10^5^ to 10^7^ PFU/mL). The control groups were only inoculated with bacteria (bacterial control) and phage (phage control), respectively. The Erlenmeyer flasks were incubated at 25 °C without agitation. Aliquots were collected at the start (T0h) and every 2 h for 12 h. The phage titre was determined in triplicate by the double-layer agar method after an incubation period of 16 to 18 h at 25 °C. Bacterial concentration was determined in triplicate in solid TSA medium through the Miles and Misra method [79] after an incubation period of 16–18 h at 25 °C. Three independent assays were performed for each condition.

### 4.9. Rate of Emergence of Phage-Resistant Bacterial Mutants

The rate of emergence of phage-resistant bacterial mutants was assessed according to Filippov et al. [80]. Briefly, from a plate of phage-sensitive bacteria, 10 isolated colonies were selected and inoculated in 10 tubes with 5 mL of TSB medium 3% NaCl and 1mM of Ca^2+^ and incubated at 25 °C with orbital shaking during 18 h. The bacterial cultures were serially diluted and 100 µL aliquots of the 10^0^ to 10^−2^ dilutions and 100 µL of concentrated phage stock (10^10^ PFU/mL) were inoculated in molten top agar (TSB 0.6% agar) and poured over TSA plates. The plates were incubated at 25 °C for 3–5 days. Simultaneously, 100 µL aliquots of 10^−5^ to 10^−7^ dilutions of the bacterial culture were plated by incorporation on TSA plates without phage and incubated at 25 °C for 24 h. The frequency of phage resistance mutation was calculated by dividing the number of bacteria grown in the presence of phage (resistant mutants) by the total number of bacteria cells (grown without added phage) [80].

### 4.10. Loss of Bacterial Resistance to Phages

The loss of bacterial phage resistance was evaluated according to Duarte et al. [23]. Briefly, ten phage resistant bacterial colonies that grew in the presence of phage were selected and grown in TSB 3% NaCl medium with 3% NaCl and 1mM of Ca^2+^ at 25 °C for 18–24 h. To confirm that the cultures remained resistant to phage, a spot test was performed on each culture. Subsequently, all cultures were streak plated to isolate new colonies and incubated at 25 °C for 18–24 h. After incubation, new liquid cultures were produced from isolated colonies and a new spot test was performed. This procedure was repeated for 10 consecutive subcultures. If sensitivity to phage was detected, two more colonies would be selected and spot tested to confirm the loss of resistance.

#### Growth Curve of Phage-Resistant Bacterial Mutants

The impact of phage resistance on bacterial growth rate was determined according to Duarte et al. [23]. The phage resistant bacterial mutants from the previous experiment (see Section 4.10) and their parent strain were cultured in parallel on a 96-well plate with TSB medium 3% NaCl at 25 °C. The O.D.600 nm of the cultures was measured at 0, 2, 4, 6, 8, 10, 12 and 24 h using a microplate photometer (Multiskan FC, Thermo Fischer, Waltham, MA, USA).

### 4.11. Statistical Analysis

The statistical analysis of data was performed using the GraphPad Prism software 6.01, San Diego, CA, USA. Normal distribution of data was confirmed using a Kolmogorov–Smirnov’s test, while homogeneity of variance was assessed using a Levene’s test. The significance of differences recorded on bacterial and viral concentrations between treatments and during the experiments was tested using a two-way ANOVA with repeated measures and Tukey’s multiple comparison post-hoc test (see Section 4.7). For different treatments, the significance of differences was evaluated by comparing the result obtained in the test samples with the results obtained for the correspondent control samples, over the different times monitored. Two-way ANOVAs were also used to analyse the statistical differences between the growth curves of the sensitive and the phage-resistant bacteria during different sampling times (Section 4.7). A value of *p* < 0.05 was considered to be statistically significant.

## 5. Conclusions

This study provides valuable insights that will guide future investigations into the application of these bacteriophages in different scenarios to control, decontaminate or treat bacterial infections or contaminations by *V. parahaemolyticus*. All four isolated phages were able to effectively reduce and control bacterial growth with very low rates of bacterial resistance. In the future, experimental evolution and adaptation of these phages may result in new phages with a broader host range, thus leading to a larger applicability in aquaculture and/or depuration units. Furthermore, studies in the loss of bacterial fitness should also be performed to better understand the impact of phage resistance.

## Figures and Tables

**Table 1:** Bacteriophage host range using 26 different bacterial strains. A clear lysis zone was considered a positive infection (+) and the absence of any clearing in the bacterial lawn was considered negative (−).

	Bacteriophage			
Bacterial Strain	S1	VPP	SH	H
*Vibrio parahaemolyticus* O22C	+	+	+	+
*Vibrio* VL06	−	−	−	−
*Vibrio* VL018	−	−	−	−
*Vibrio* CF02	−	−	−	−
*Vibrio* CF03	−	−	−	−
*Vibrio* CF06	−	−	−	−
*Vibrio parahaemolyticus* V9A	−	−	−	−
*Vibrio parahaemolyticus* V9B	−	−	−	−
*Vibrio parahaemolyticus* V10B	−	−	−	−
*Vibrio alginolyticus* CECT 521	−	−	−	−
*Vibrio alginolyticus* ARG 636.1	−	−	−	−
*Vibrio alginolyticus* ARG 625.2	−	−	−	−
*Vibrio alginolyticus* ARG 428.1	−	−	−	−
*Vibrio harveyi* APP 75.1	−	−	−	−
*Vibrio alginolyticus AQV 47.1*	−	−	−	−
*Vibrio europaeus L4*	−	−	−	−
*Vibrio vulnificus* ATCC 19.1	−	−	−	−
*Vibrio vulnificus* ATCC 27562	−	−	−	−
*Vibrio parahaemolyticus* DSM 27657	−	−	−	−
*Vibrio parahaemolyticus* ATCC 17802	−	−	−	−
*Aliivibrio fisheri* ATCC 49387	−	−	−	−
*Photobacterium damselae damselae* DSM 7482	−	−	−	−
*Aeromonas hydrophila* ATCC 7966	−	−	−	−
*Aeromonas salmonicida* CECT 894	−	−	−	−
*Escherichia coli* ATCC 13706	−	−	−	−
*Pseudomonas aeruginosa* ATCC 27853	−	−	−	−

**Table 2:** Genomic features of the phages isolated in present study.

Phage Name	S1	VPP	SH	H
Source	Water	Water	Water	Water
Isolation host	*V. parahaemolyticus*	*V. parahaemolyticus*	*V. parahaemolyticus*	*V. parahaemolyticus*
Classification	*Caudoviricetes*	*Caudoviricetes*	*Caudoviricetes*	*Caudoviricetes*
Genome size (bp)	44,893	44,890	45,137	44,967
GC content (%)	43.43	43.43	43.45	43.44
Completeness (%)	100	100	100	100
Contamination (%)	0	0	0	0
No. of CDS	66	66	67	66
No. of hypothetical proteins	42 (64%)	42 (64%)	45 (67%)	43 (65%)
tRNA genes	0	0	0	0
Accession number	PQ284949	PQ284950	PQ284951	PQ284952
Closest database relative according to BLASTn	no hit	no hit	no hit	no hit

**Table 4:** Number of consecutive passages and the resistance loss through spot test analysis for each isolated bacterium (R1–R10). (−) bacterial culture remained phage resistant; (+) bacterial culture lost phage resistance. Numbers represent the number of positive results on a total of three tests.

Consecutive Passage	R1	R2	R3	R4	R5	R6	R7	R8	R9	R10
1	−	−	−	−	−	−	−	−	−	−
2	−	−	−	−	−	−	−	−	−	−
3	−	−	−	−	−	−	−	−	−	−
4	−	−	−	−	−	−	−	−	−	−
5	−	−	−	−	−	−	−	−	−	−
6	−	−	−	−	−	−	−	−	−	−
7	−	−	−	−	−	−	−	−	−	−
8	−	−	−	−	−	−	−	−	−	−
9	−	−	−	−	−	−	−	−	−	−
10	−	−	−	−	−	−	−	−	− (2/3)	+ (3/3)

**Table 5:** Test groups and characteristics of each group used in the evaluation of calcium and magnesium requirements for phage replication.

Group Name	Group Characteristics
Phage control PBS	PBS + phage
Phage control TSB	TSB 3% NaCl + phage
Phage Infection with no cations	TSB 3% NaCl + phage + bacteria
Phage Infection with calcium	TSB 3% NaCl + Ca^2+^ + phage + bacteria
Phage Infection with magnesium	TSB 3% NaCl + Mg^2+^ + phage + bacteria
Phage Infection with both cations	TSB 3% NaCl + Ca^2+^ + Mg^2+^ + phage + bacteria

## Data Availability

Data are contained within the article and Appendix A and Appendix B.

## Appendix A

Genome analysis of the four phages.

**Table A1:** Coding sequences identified in S1 phage.

Gene	Start	Stop	Frame	Region	Putative Function	PHROGs Category
FWOTDMFH_CDS_0001	525	1	−	CDS	hypothetical protein	unknown function
FWOTDMFH_CDS_0002	888	1103	+	CDS	hypothetical protein	unknown function
FWOTDMFH_CDS_0003	1090	1494	+	CDS	HNH endonuclease	DNA, RNA and nucleotide metabolism
FWOTDMFH_CDS_0004	1909	2187	+	CDS	hypothetical protein	unknown function
FWOTDMFH_CDS_0005	2147	2440	+	CDS	hypothetical protein	unknown function
FWOTDMFH_CDS_0006	2443	2631	+	CDS	hypothetical protein	unknown function
FWOTDMFH_CDS_0007	2628	2876	+	CDS	hypothetical protein	unknown function
FWOTDMFH_CDS_0008	2987	3178	+	CDS	hypothetical protein	unknown function
FWOTDMFH_CDS_0009	3195	3437	+	CDS	hypothetical protein	unknown function
FWOTDMFH_CDS_0010	3767	4159	+	CDS	hypothetical protein	unknown function
FWOTDMFH_CDS_0011	4180	4584	+	CDS	hypothetical protein	unknown function
FWOTDMFH_CDS_0012	4581	6383	+	CDS	terminase large subunit	head and packaging
FWOTDMFH_CDS_0013	6396	6560	+	CDS	hypothetical protein	unknown function
FWOTDMFH_CDS_0014	6602	7882	+	CDS	portal protein	head and packaging
FWOTDMFH_CDS_0015	7872	8522	+	CDS	head maturation protease	head and packaging
FWOTDMFH_CDS_0016	8609	9988	+	CDS	major head protein	head and packaging
FWOTDMFH_CDS_0017	10,095	10,610	+	CDS	head–tail adaptor	connector
FWOTDMFH_CDS_0018	10,603	10,923	+	CDS	head closure Hc1	connector
FWOTDMFH_CDS_0019	10,920	11,489	+	CDS	hypothetical protein	unknown function
FWOTDMFH_CDS_0020	11,516	11,845	+	CDS	hypothetical protein	unknown function
FWOTDMFH_CDS_0021	11,857	12,534	+	CDS	major tail protein	tail
FWOTDMFH_CDS_0022	12,591	12,974	+	CDS	hypothetical protein	unknown function
FWOTDMFH_CDS_0023	13,177	13,710	+	CDS	hypothetical protein	unknown function
FWOTDMFH_CDS_0024	14,124	13,690	−	CDS	hypothetical protein	unknown function
FWOTDMFH_CDS_0025	14,227	16,554	+	CDS	tail length tape measure protein	tail
FWOTDMFH_CDS_0026	16,760	16,575	−	CDS	hypothetical protein	unknown function
FWOTDMFH_CDS_0027	16,859	17,350	+	CDS	virion structural protein	head and packaging
FWOTDMFH_CDS_0028	17,347	17,862	+	CDS	hypothetical protein	unknown function
FWOTDMFH_CDS_0029	17,859	18,206	+	CDS	hypothetical protein	unknown function
FWOTDMFH_CDS_0030	18,194	20,929	+	CDS	tail protein	tail
FWOTDMFH_CDS_0031	20,926	23,181	+	CDS	tail protein	tail
FWOTDMFH_CDS_0032	23,956	23,294	−	CDS	DNA polymerase	DNA, RNA and nucleotide metabolism
FWOTDMFH_CDS_0033	25,385	23,961	−	CDS	DNA polymerase	DNA, RNA and nucleotide metabolism
FWOTDMFH_CDS_0034	25,567	25,382	−	CDS	hypothetical protein	unknown function
FWOTDMFH_CDS_0035	25,746	25,549	−	CDS	hypothetical protein	unknown function
FWOTDMFH_CDS_0036	26,152	25,721	−	CDS	hypothetical protein	unknown function
FWOTDMFH_CDS_0037	27,444	26,149	−	CDS	hypothetical protein	unknown function
FWOTDMFH_CDS_0038	27,681	27,529	−	CDS	hypothetical protein	unknown function
FWOTDMFH_CDS_0039	30,160	27,695	−	CDS	DNA polymerase/primase	DNA, RNA and nucleotide metabolism
FWOTDMFH_CDS_0040	30,406	30,170	−	CDS	hypothetical protein	unknown function
FWOTDMFH_CDS_0041	30,822	30,403	−	CDS	hypothetical protein	unknown function
FWOTDMFH_CDS_0042	31,260	30,823	−	CDS	exonuclease recombination-associated	DNA, RNA and nucleotide metabolism
FWOTDMFH_CDS_0043	31,681	31,358	−	CDS	exonuclease recombination-associated	DNA, RNA and nucleotide metabolism
FWOTDMFH_CDS_0044	32,112	31,756	−	CDS	exonuclease recombination-associated	DNA, RNA and nucleotide metabolism
FWOTDMFH_CDS_0045	32,681	32,193	−	CDS	exonuclease recombination-associated	DNA, RNA and nucleotide metabolism
FWOTDMFH_CDS_0046	33,872	32,742	−	CDS	hypothetical protein	unknown function
FWOTDMFH_CDS_0047	34,258	33,887	−	CDS	MazG-like pyrophosphatase	other
FWOTDMFH_CDS_0048	35,387	34,266	−	CDS	exonuclease	DNA, RNA and nucleotide metabolism
FWOTDMFH_CDS_0049	36,298	35,393	−	CDS	hypothetical protein	unknown function
FWOTDMFH_CDS_0050	36,687	36,947	+	CDS	hypothetical protein	unknown function
FWOTDMFH_CDS_0051	36,944	38,641	+	CDS	DNA helicase	DNA, RNA and nucleotide metabolism
FWOTDMFH_CDS_0052	38,638	38,868	+	CDS	hypothetical protein	unknown function
FWOTDMFH_CDS_0053	38,858	39,034	+	CDS	hypothetical protein	unknown function
FWOTDMFH_CDS_0054	39,021	39,167	+	CDS	hypothetical protein	unknown function
FWOTDMFH_CDS_0055	39,157	39,618	+	CDS	amidase	lysis
FWOTDMFH_CDS_0056	39,621	39,809	+	CDS	hypothetical protein	unknown function
FWOTDMFH_CDS_0057	39,802	40,080	+	CDS	hypothetical protein	unknown function
FWOTDMFH_CDS_0058	40,990	40,721	−	CDS	hypothetical protein	unknown function
FWOTDMFH_CDS_0059	41,305	41,081	−	CDS	hypothetical protein	unknown function
FWOTDMFH_CDS_0060	41,557	41,309	−	CDS	hypothetical protein	unknown function
FWOTDMFH_CDS_0061	41,801	41,625	−	CDS	hypothetical protein	unknown function
FWOTDMFH_CDS_0062	42,241	41,897	−	CDS	hypothetical protein	unknown function
FWOTDMFH_CDS_0063	42,591	42,238	−	CDS	hypothetical protein	unknown function
FWOTDMFH_CDS_0064	43,025	42,594	−	CDS	hypothetical protein	unknown function
FWOTDMFH_CDS_0065	44,357	43,308	−	CDS	DNA methyltransferase	other
FWOTDMFH_CDS_0066	44,893	44,354	−	CDS	hypothetical protein	unknown function

**Table A2:** Coding sequences identified in VPP phage.

Gene	Start	Stop	Frame	Region	Putative Function	PHROGs Category
WKREMNYY_CDS_0001	1	429	+	CDS	major head protein	head and packaging
WKREMNYY_CDS_0002	536	1051	+	CDS	head–tail adaptor	connector
WKREMNYY_CDS_0003	1044	1364	+	CDS	head closure Hc1	connector
WKREMNYY_CDS_0004	1361	1930	+	CDS	hypothetical protein	unknown function
WKREMNYY_CDS_0005	1957	2286	+	CDS	hypothetical protein	unknown function
WKREMNYY_CDS_0006	2298	2975	+	CDS	major tail protein	tail
WKREMNYY_CDS_0007	3032	3415	+	CDS	hypothetical protein	unknown function
WKREMNYY_CDS_0008	3618	4151	+	CDS	hypothetical protein	unknown function
WKREMNYY_CDS_0009	4565	4131	−	CDS	hypothetical protein	unknown function
WKREMNYY_CDS_0010	4668	6995	+	CDS	tail length tape measure protein	tail
WKREMNYY_CDS_0011	7300	7791	+	CDS	virion structural protein	head and packaging
WKREMNYY_CDS_0012	7788	8303	+	CDS	hypothetical protein	unknown function
WKREMNYY_CDS_0013	8300	8647	+	CDS	hypothetical protein	unknown function
WKREMNYY_CDS_0014	8635	11,370	+	CDS	tail protein	tail
WKREMNYY_CDS_0015	11,367	13,622	+	CDS	tail protein	tail
WKREMNYY_CDS_0016	14,397	13,735	−	CDS	DNA polymerase	DNA, RNA and nucleotide metabolism
WKREMNYY_CDS_0017	15,826	14,402	−	CDS	DNA polymerase	DNA, RNA and nucleotide metabolism
WKREMNYY_CDS_0018	16,008	15,823	−	CDS	hypothetical protein	unknown function
WKREMNYY_CDS_0019	16,187	15,990	−	CDS	hypothetical protein	unknown function
WKREMNYY_CDS_0020	16,593	16,162	−	CDS	hypothetical protein	unknown function
WKREMNYY_CDS_0021	17,885	16,590	−	CDS	hypothetical protein	unknown function
WKREMNYY_CDS_0022	18,122	17,970	−	CDS	hypothetical protein	unknown function
WKREMNYY_CDS_0023	20,601	18,136	−	CDS	DNA polymerase/primase	DNA, RNA and nucleotide metabolism
WKREMNYY_CDS_0024	20,847	20,611	−	CDS	hypothetical protein	unknown function
WKREMNYY_CDS_0025	21,263	20,844	−	CDS	hypothetical protein	unknown function
WKREMNYY_CDS_0026	21,701	21,264	−	CDS	exonuclease recombination-associated	DNA, RNA and nucleotide metabolism
WKREMNYY_CDS_0027	22,119	21,799	−	CDS	exonuclease recombination-associated	DNA, RNA and nucleotide metabolism
WKREMNYY_CDS_0028	22,547	22,194	−	CDS	exonuclease recombination-associated	DNA, RNA and nucleotide metabolism
WKREMNYY_CDS_0029	23,119	22,628	−	CDS	exonuclease recombination-associated	DNA, RNA and nucleotide metabolism
WKREMNYY_CDS_0030	24,310	23,180	−	CDS	hypothetical protein	unknown function
WKREMNYY_CDS_0031	24,696	24,325	−	CDS	MazG-like pyrophosphatase	other
WKREMNYY_CDS_0032	25,825	24,704	−	CDS	exonuclease	DNA, RNA and nucleotide metabolism
WKREMNYY_CDS_0033	26,736	25,831	−	CDS	hypothetical protein	unknown function
WKREMNYY_CDS_0034	26,970	27,137	+	CDS	hypothetical protein	unknown function
WKREMNYY_CDS_0035	27,125	27,385	+	CDS	hypothetical protein	unknown function
WKREMNYY_CDS_0036	27,382	29,079	+	CDS	DNA helicase	DNA, RNA and nucleotide metabolism
WKREMNYY_CDS_0037	29,076	29,306	+	CDS	hypothetical protein	unknown function
WKREMNYY_CDS_0038	29,296	29,472	+	CDS	hypothetical protein	unknown function
WKREMNYY_CDS_0039	29,459	29,605	+	CDS	hypothetical protein	unknown function
WKREMNYY_CDS_0040	29,595	30,056	+	CDS	amidase	lysis
WKREMNYY_CDS_0041	30,059	30,247	+	CDS	hypothetical protein	unknown function
WKREMNYY_CDS_0042	30,240	30,518	+	CDS	hypothetical protein	unknown function
WKREMNYY_CDS_0043	31,428	31,159	−	CDS	hypothetical protein	unknown function
WKREMNYY_CDS_0044	31,743	31,519	−	CDS	hypothetical protein	unknown function
WKREMNYY_CDS_0045	31,995	31,747	−	CDS	hypothetical protein	unknown function
WKREMNYY_CDS_0046	32,239	32,063	−	CDS	hypothetical protein	unknown function
WKREMNYY_CDS_0047	32,679	32,335	−	CDS	hypothetical protein	unknown function
WKREMNYY_CDS_0048	33,029	32,676	−	CDS	hypothetical protein	unknown function
WKREMNYY_CDS_0049	33,463	33,032	−	CDS	hypothetical protein	unknown function
WKREMNYY_CDS_0050	34,795	33,746	−	CDS	DNA methyltransferase	other
WKREMNYY_CDS_0051	35,856	34,792	−	CDS	hypothetical protein	unknown function
WKREMNYY_CDS_0052	36,219	36,434	+	CDS	hypothetical protein	unknown function
WKREMNYY_CDS_0053	36,421	36,825	+	CDS	HNH endonuclease	DNA, RNA and nucleotide metabolism
WKREMNYY_CDS_0054	37,240	37,518	+	CDS	hypothetical protein	unknown function
WKREMNYY_CDS_0055	37,478	37,771	+	CDS	hypothetical protein	unknown function
WKREMNYY_CDS_0056	37,774	37,962	+	CDS	hypothetical protein	unknown function
WKREMNYY_CDS_0057	37,959	38,207	+	CDS	hypothetical protein	unknown function
WKREMNYY_CDS_0058	38,318	38,509	+	CDS	hypothetical protein	unknown function
WKREMNYY_CDS_0059	38,526	38,768	+	CDS	hypothetical protein	unknown function
WKREMNYY_CDS_0060	39,098	39,490	+	CDS	hypothetical protein	unknown function
WKREMNYY_CDS_0061	39,511	39,915	+	CDS	hypothetical protein	unknown function
WKREMNYY_CDS_0062	39,912	41,714	+	CDS	terminase large subunit	head and packaging
WKREMNYY_CDS_0063	41,727	41,891	+	CDS	hypothetical protein	unknown function
WKREMNYY_CDS_0064	41,933	43,213	+	CDS	portal protein	head and packaging
WKREMNYY_CDS_0065	43,203	43,853	+	CDS	head maturation protease	head and packaging
WKREMNYY_CDS_0066	43,940	44,890	+	CDS	hypothetical protein	unknown function

**Table A3:** Coding sequences identified in SH phage.

Gene	Start	Stop	Frame	Region	Putative Function	PHROGs Category
UYWVGLBB_CDS_0001	146	3	−	CDS	hypothetical protein	unknown function
UYWVGLBB_CDS_0002	645	169	−	CDS	head–tail adaptor	connector
UYWVGLBB_CDS_0003	2131	752	−	CDS	major head protein	head and packaging
UYWVGLBB_CDS_0004	2868	2218	−	CDS	head maturation protease	head and packaging
UYWVGLBB_CDS_0005	4138	2858	−	CDS	portal protein	head and packaging
UYWVGLBB_CDS_0006	4344	4180	−	CDS	hypothetical protein	unknown function
UYWVGLBB_CDS_0007	6159	4357	−	CDS	terminase large subunit	head and packaging
UYWVGLBB_CDS_0008	6560	6156	−	CDS	hypothetical protein	unknown function
UYWVGLBB_CDS_0009	6973	6581	−	CDS	hypothetical protein	unknown function
UYWVGLBB_CDS_0010	7545	7303	−	CDS	hypothetical protein	unknown function
UYWVGLBB_CDS_0011	7753	7562	−	CDS	hypothetical protein	unknown function
UYWVGLBB_CDS_0012	8112	7864	−	CDS	hypothetical protein	unknown function
UYWVGLBB_CDS_0013	8297	8109	−	CDS	hypothetical protein	unknown function
UYWVGLBB_CDS_0014	8536	8300	−	CDS	hypothetical protein	unknown function
UYWVGLBB_CDS_0015	8831	8553	−	CDS	hypothetical protein	unknown function
UYWVGLBB_CDS_0016	9650	9246	−	CDS	HNH endonuclease	DNA, RNA and nucleotide metabolism
UYWVGLBB_CDS_0017	9852	9637	−	CDS	hypothetical protein	unknown function
UYWVGLBB_CDS_0018	10,215	11,279	+	CDS	hypothetical protein	unknown function
UYWVGLBB_CDS_0019	11,276	12,325	+	CDS	DNA methyltransferase	other
UYWVGLBB_CDS_0020	12,608	13,039	+	CDS	hypothetical protein	unknown function
UYWVGLBB_CDS_0021	13,042	13,395	+	CDS	hypothetical protein	unknown function
UYWVGLBB_CDS_0022	13,392	13,736	+	CDS	hypothetical protein	unknown function
UYWVGLBB_CDS_0023	13,832	14,008	+	CDS	hypothetical protein	unknown function
UYWVGLBB_CDS_0024	13,998	14,324	+	CDS	hypothetical protein	unknown function
UYWVGLBB_CDS_0025	14,328	14,552	+	CDS	hypothetical protein	unknown function
UYWVGLBB_CDS_0026	14,643	14,912	+	CDS	hypothetical protein	unknown function
UYWVGLBB_CDS_0027	15,831	15,553	−	CDS	hypothetical protein	unknown function
UYWVGLBB_CDS_0028	16,012	15,824	−	CDS	hypothetical protein	unknown function
UYWVGLBB_CDS_0029	16,476	16,015	−	CDS	amidase	lysis
UYWVGLBB_CDS_0030	16,612	16,466	−	CDS	hypothetical protein	unknown function
UYWVGLBB_CDS_0031	16,775	16,599	−	CDS	hypothetical protein	unknown function
UYWVGLBB_CDS_0032	16,995	16,765	−	CDS	hypothetical protein	unknown function
UYWVGLBB_CDS_0033	18,689	16,992	−	CDS	DNA helicase	DNA, RNA and nucleotide metabolism
UYWVGLBB_CDS_0034	18,946	18,686	−	CDS	hypothetical protein	unknown function
UYWVGLBB_CDS_0035	19,165	18,953	−	CDS	hypothetical protein	unknown function
UYWVGLBB_CDS_0036	19,335	20,240	+	CDS	hypothetical protein	unknown function
UYWVGLBB_CDS_0037	20,246	21,367	+	CDS	exonuclease	DNA, RNA and nucleotide metabolism
UYWVGLBB_CDS_0038	21,375	21,746	+	CDS	MazG-like pyrophosphatase	other
UYWVGLBB_CDS_0039	21,761	22,891	+	CDS	hypothetical protein	unknown function
UYWVGLBB_CDS_0040	22,952	23,782	+	CDS	exonuclease recombination-associated	DNA, RNA and nucleotide metabolism
UYWVGLBB_CDS_0041	23,779	23,892	+	CDS	hypothetical protein	unknown function
UYWVGLBB_CDS_0042	23,953	24,366	+	CDS	exonuclease recombination-associated	DNA, RNA and nucleotide metabolism
UYWVGLBB_CDS_0043	24,447	24,884	+	CDS	exonuclease recombination-associated	DNA, RNA and nucleotide metabolism
UYWVGLBB_CDS_0044	24,885	25,304	+	CDS	hypothetical protein	unknown function
UYWVGLBB_CDS_0045	25,301	25,537	+	CDS	hypothetical protein	unknown function
UYWVGLBB_CDS_0046	25,547	28,012	+	CDS	DNA polymerase/primase	DNA, RNA and nucleotide metabolism
UYWVGLBB_CDS_0047	28,026	28,178	+	CDS	hypothetical protein	unknown function
UYWVGLBB_CDS_0048	28,263	29,558	+	CDS	hypothetical protein	unknown function
UYWVGLBB_CDS_0049	29,555	29,986	+	CDS	hypothetical protein	unknown function
UYWVGLBB_CDS_0050	29,961	30,158	+	CDS	hypothetical protein	unknown function
UYWVGLBB_CDS_0051	30,140	30,325	+	CDS	hypothetical protein	unknown function
UYWVGLBB_CDS_0052	30,322	31,746	+	CDS	DNA polymerase	DNA, RNA and nucleotide metabolism
UYWVGLBB_CDS_0053	31,751	32,413	+	CDS	DNA polymerase	DNA, RNA and nucleotide metabolism
UYWVGLBB_CDS_0054	34,781	32,526	−	CDS	tail protein	tail
UYWVGLBB_CDS_0055	37,513	34,778	−	CDS	tail protein	tail
UYWVGLBB_CDS_0056	37,848	37,501	−	CDS	hypothetical protein	unknown function
UYWVGLBB_CDS_0057	38,360	37,845	−	CDS	hypothetical protein	unknown function
UYWVGLBB_CDS_0058	38,848	38,357	−	CDS	virion structural protein	head and packaging
UYWVGLBB_CDS_0059	41,480	39,153	−	CDS	tail length tape measure protein	tail
UYWVGLBB_CDS_0060	41,583	42,017	+	CDS	hypothetical protein	unknown function
UYWVGLBB_CDS_0061	42,530	41,997	−	CDS	hypothetical protein	unknown function
UYWVGLBB_CDS_0062	43,116	42,733	−	CDS	hypothetical protein	unknown function
UYWVGLBB_CDS_0063	43,850	43,173	−	CDS	major tail protein	tail
UYWVGLBB_CDS_0064	44,221	43,862	−	CDS	hypothetical protein	unknown function
UYWVGLBB_CDS_0065	44,787	44,218	−	CDS	hypothetical protein	unknown function
UYWVGLBB_CDS_0066	45,017	44,784	−	CDS	hypothetical protein	unknown function
UYWVGLBB_CDS_0067	45,111	44,977	−	CDS	hypothetical protein	unknown function

**Table A4:** Coding sequences identified in H phage.

Gene	Start	Stop	Frame	Region	Putative Function	PHROGs Category
NVEJSIEB_CDS_0001	525	1	−	CDS	hypothetical protein	unknown function
NVEJSIEB_CDS_0002	888	1103	+	CDS	hypothetical protein	unknown function
NVEJSIEB_CDS_0003	1090	1494	+	CDS	HNH endonuclease	DNA, RNA and nucleotide metabolism
NVEJSIEB_CDS_0004	1909	2187	+	CDS	hypothetical protein	unknown function
NVEJSIEB_CDS_0005	2204	2440	+	CDS	hypothetical protein	unknown function
NVEJSIEB_CDS_0006	2443	2631	+	CDS	hypothetical protein	unknown function
NVEJSIEB_CDS_0007	2628	2876	+	CDS	hypothetical protein	unknown function
NVEJSIEB_CDS_0008	2987	3178	+	CDS	hypothetical protein	unknown function
NVEJSIEB_CDS_0009	3195	3437	+	CDS	hypothetical protein	unknown function
NVEJSIEB_CDS_0010	3767	4159	+	CDS	hypothetical protein	unknown function
NVEJSIEB_CDS_0011	4180	4584	+	CDS	hypothetical protein	unknown function
NVEJSIEB_CDS_0012	4581	6383	+	CDS	terminase large subunit	head and packaging
NVEJSIEB_CDS_0013	6396	6560	+	CDS	hypothetical protein	unknown function
NVEJSIEB_CDS_0014	6602	7882	+	CDS	portal protein	head and packaging
NVEJSIEB_CDS_0015	7872	8522	+	CDS	head maturation protease	head and packaging
NVEJSIEB_CDS_0016	8609	9988	+	CDS	major head protein	head and packaging
NVEJSIEB_CDS_0017	10,095	10,610	+	CDS	head–tail adaptor	connector
NVEJSIEB_CDS_0018	10,603	10,923	+	CDS	head closure Hc1	connector
NVEJSIEB_CDS_0019	10,920	11,489	+	CDS	hypothetical protein	unknown function
NVEJSIEB_CDS_0020	11,486	11,845	+	CDS	hypothetical protein	unknown function
NVEJSIEB_CDS_0021	11,857	12,534	+	CDS	major tail protein	tail
NVEJSIEB_CDS_0022	12,591	12,974	+	CDS	hypothetical protein	unknown function
NVEJSIEB_CDS_0023	13,177	13,710	+	CDS	hypothetical protein	unknown function
NVEJSIEB_CDS_0024	14,124	13,690	−	CDS	hypothetical protein	unknown function
NVEJSIEB_CDS_0025	14,227	16,554	+	CDS	tail length tape measure protein	tail
NVEJSIEB_CDS_0026	16,760	16,575	−	CDS	hypothetical protein	unknown function
NVEJSIEB_CDS_0027	16,859	17,350	+	CDS	virion structural protein	head and packaging
NVEJSIEB_CDS_0028	17,347	17,862	+	CDS	hypothetical protein	unknown function
NVEJSIEB_CDS_0029	17,859	18,206	+	CDS	hypothetical protein	unknown function
NVEJSIEB_CDS_0030	18,194	20,929	+	CDS	tail protein	tail
NVEJSIEB_CDS_0031	20,926	23,181	+	CDS	tail protein	tail
NVEJSIEB_CDS_0032	23,956	23,294	−	CDS	DNA polymerase	DNA, RNA and nucleotide metabolism
NVEJSIEB_CDS_0033	25,385	23,961	−	CDS	DNA polymerase	DNA, RNA and nucleotide metabolism
NVEJSIEB_CDS_0034	25,567	25,382	−	CDS	hypothetical protein	unknown function
NVEJSIEB_CDS_0035	25,746	25,549	−	CDS	hypothetical protein	unknown function
NVEJSIEB_CDS_0036	26,152	25,721	−	CDS	hypothetical protein	unknown function
NVEJSIEB_CDS_0037	27,444	26,149	−	CDS	hypothetical protein	unknown function
NVEJSIEB_CDS_0038	27,681	27,529	−	CDS	hypothetical protein	unknown function
NVEJSIEB_CDS_0039	30,160	27,695	−	CDS	DNA polymerase/primase	DNA, RNA and nucleotide metabolism
NVEJSIEB_CDS_0040	30,406	30,170	−	CDS	hypothetical protein	unknown function
NVEJSIEB_CDS_0041	30,822	30,403	−	CDS	hypothetical protein	unknown function
NVEJSIEB_CDS_0042	31,755	30,823	−	CDS	exonuclease recombination-associated	DNA, RNA and nucleotide metabolism
NVEJSIEB_CDS_0043	31,929	31,816	−	CDS	hypothetical protein	unknown function
NVEJSIEB_CDS_0044	32,264	31,926	−	CDS	exonuclease recombination-associated	DNA, RNA and nucleotide metabolism
NVEJSIEB_CDS_0045	32,755	32,345	−	CDS	exonuclease recombination-associated	DNA, RNA and nucleotide metabolism
NVEJSIEB_CDS_0046	33,946	32,816	−	CDS	hypothetical protein	unknown function
NVEJSIEB_CDS_0047	34,332	33,961	−	CDS	MazG-like pyrophosphatase	other
NVEJSIEB_CDS_0048	35,461	34,340	−	CDS	exonuclease	DNA, RNA and nucleotide metabolism
NVEJSIEB_CDS_0049	36,372	35,467	−	CDS	hypothetical protein	unknown function
NVEJSIEB_CDS_0050	36,761	37,021	+	CDS	hypothetical protein	unknown function
NVEJSIEB_CDS_0051	37,018	38,715	+	CDS	DNA helicase	DNA, RNA and nucleotide metabolism
NVEJSIEB_CDS_0052	38,712	38,942	+	CDS	hypothetical protein	unknown function
NVEJSIEB_CDS_0053	38,932	39,108	+	CDS	hypothetical protein	unknown function
NVEJSIEB_CDS_0054	39,095	39,241	+	CDS	hypothetical protein	unknown function
NVEJSIEB_CDS_0055	39,231	39,692	+	CDS	amidase	lysis
NVEJSIEB_CDS_0056	39,695	39,883	+	CDS	hypothetical protein	unknown function
NVEJSIEB_CDS_0057	39,876	40,154	+	CDS	hypothetical protein	unknown function
NVEJSIEB_CDS_0058	41,064	40,795	−	CDS	hypothetical protein	unknown function
NVEJSIEB_CDS_0059	41,379	41,155	−	CDS	hypothetical protein	unknown function
NVEJSIEB_CDS_0060	41,709	41,383	−	CDS	hypothetical protein	unknown function
NVEJSIEB_CDS_0061	41,875	41,699	−	CDS	hypothetical protein	unknown function
NVEJSIEB_CDS_0062	42,315	41,971	−	CDS	hypothetical protein	unknown function
NVEJSIEB_CDS_0063	42,665	42,312	−	CDS	hypothetical protein	unknown function
NVEJSIEB_CDS_0064	43,099	42,668	−	CDS	hypothetical protein	unknown function
NVEJSIEB_CDS_0065	44,431	43,382	−	CDS	DNA methyltransferase	other
NVEJSIEB_CDS_0066	44,967	44,428	−	CDS	hypothetical protein	unknown function

## Appendix B

Inactivation curve of bacteria in the presence of the four phages individually in standard TSB media and in TSB supplemented with Calcium 1 mM.
